# Deletion of the stress response protein REDD1 prevents sodium iodate-induced RPE damage and photoreceptor loss

**DOI:** 10.1007/s11357-024-01362-2

**Published:** 2024-10-05

**Authors:** Sandeep M. Subrahmanian, Esma I. Yerlikaya, Siddharth Sunilkumar, Allyson L. Toro, Christopher M. McCurry, Stephanie L. Grillo, Alistair J. Barber, Jeffrey M. Sundstrom, Michael D. Dennis

**Affiliations:** 1 Department of Cellular and Molecular Physiology, Penn State College of Medicine, 500 University Drive, Hershey, PA 17033 USA; 2 Department of Ophthalmology, Penn State College of Medicine, Hershey, PA 17033 USA

**Keywords:** DDIT4, RTP801, Retinal pigmented epithelium, Aging

## Abstract

Age-related macular degeneration (AMD) is a leading cause of blindness in elderly populations, yet the molecular events that initiate the early retinal defects that lead to visual function deficits remain poorly understood. The studies here explored a role for the stress response protein Regulated in Development and DNA damage response 1 (REDD1) in the development of retinal pathology by using the oxidant stressor sodium iodate (NaIO_3_) to model dry AMD in mice. REDD1 protein abundance was increased in the retinal pigmented epithelium (RPE) and retina of mice administered NaIO_3_. In wild-type REDD1^+/+^ mice, reactive oxygen species (ROS) levels were robustly increased in the outer retinal layers 1 day after NaIO_3_ administration, with focal areas of increased ROS seen throughout the outer retina after 7 days. In contrast with REDD1^+/+^ mice, ROS levels were blunted in REDD1^−/−^ mice after NaIO_3_ administration. REDD1 was also required for upregulated expression of pro-inflammatory factors in the RPE/retina and immune cell activation in the outer retina following NaIO_3_ administration. In REDD1^+/+^ mice, NaIO_3_ reduced RPE65 and rhodopsin levels in the RPE and photoreceptor layers, respectively. Unlike REDD1^+/+^ mice, REDD1^−/−^ mice did not exhibit disrupted RPE integrity, retinal degeneration, or photoreceptor thinning. Overall, REDD1 deletion was sufficient to prevent retinal oxidative stress, RPE damage, immune cell activation, and photoreceptor loss in response to NaIO_3_. The findings support a potential role for REDD1 in the development of retinal complications in the context of dry AMD.

**Supplementary Information:**

The online version contains supplementary material available at 10.1007/s11357-024-01362-2.

## Introduction

Age-related macular degeneration (AMD) is the leading cause of irreversible vision loss among the elderly. Globally, AMD affects around 8.7% of the population, with an estimated 288 million individuals projected to be affected by 2040 [1]. AMD can be classified into dry and wet forms based on distinct pathological features. Dry AMD constitutes about 90% of AMD cases and involves the gradual degeneration of the retinal pigment epithelium (RPE) and photoreceptors in the macula [2]. Currently, no effective treatments are available for dry AMD, emphasizing the importance of investigations into its mechanisms and the identification of potential therapeutic targets.

RPE damage is a critical component of AMD [3]. RPE cells phagocytose the photoreceptor outer segments and play a major role in maintaining the integrity of the retina [4, 5]. Moreover, RPE cells transport nutrients and metabolites to the retina, store and recycle retinoids, and contribute to retinal homeostasis [6, 7]. Thus, defects within RPE can result in photoreceptor degeneration and lead to AMD [8]. Various factors, including aging, oxidative stress, chronic inflammation, and genetic abnormalities, are known to contribute to RPE dysfunction and macular degeneration. Among these, oxidative stress-induced damage to the RPE is considered the primary pathological mechanism underlying AMD [9–11].

The stress response protein Regulated in Development and DNA damage 1 (REDD1, also known as RTP801/DDIT4) has been implicated as a causal factor in the development of oxidative stress [12–15]. Our laboratory has shown that REDD1 protein abundance is increased in the retina in multiple preclinical models of diabetes and is necessary for diabetes-induced retinal pathology and functional deficits in vision [16–18]. A recent study demonstrated that REDD1 protein abundance is also increased in the RPE/choroid of aged mice in coordination with RPE dysfunction [19]. Recent in vitro studies also support that in ARPE-19 cells, REDD1 expression is upregulated in response to hyperglycemic conditions or the advanced-glycation end product (AGE) stimulus D-galactose concomitant with the onset of oxidative stress [19, 20]. We recently demonstrated that in addition to promoting the development of oxidative stress, REDD1 also plays a critical role in the development of retinal inflammation in diabetic mice [21].

In the present study, we explored the role of REDD1 in RPE damage, retinal degeneration, and its potential implication in AMD pathogenesis by administering the oxidant stressor sodium iodate (NaIO_3_) to mice. NaIO_3_ administration models dry AMD by inducing RPE dysfunction and macrophage accumulation, resulting in photoreceptor injury, thinning of the outer and inner segments, and reduced visual acuity [22–24]. We found that REDD1 was upregulated in the retina of mice upon administration of NaIO_3_ and that deletion of REDD1 was sufficient to prevent oxidative stress, immune cell activation, and disrupted retinal morphology. The findings suggest that REDD1 may play an important role in the development of RPE and photoreceptor damage in dry AMD.

## Methods

### Animals

Eight-week-old male and female B6;129 wild-type (REDD1^+/+^) and REDD1-deficient (REDD1^−/−^) mice [25] were maintained under 12-h light–dark conditions in a room with low light intensity (~ 325 lx at 1 m above cage floor). Mice were randomly assigned to experimental groups, and littermate controls were used whenever possible. For NaIO_3_ administration, sterile NaIO_3_ solution was freshly prepared in 0.9% saline and filtered (0.22 µm membrane). Each mouse received a single intraperitoneal injection of 20 mg/kg NaIO_3_, as previously described [26]. Control mice were injected with equivalent volumes of 0.9% saline vehicle. Eyes were collected either 1 or 7 days after NaIO_3_ administration. The selected endpoints were chosen based on prior literature [24] and a preliminary time course study (Fig. S1) demonstrating the onset of gene expression changes in the RPE and neuroretina post-NaIO_3_ administration. All animal procedures were approved by the Penn State College of Medicine Institutional Animal Care and Use Committee.

### Analysis of mRNA from RPE and retina

RNA was isolated from the RPE/choroid/sclera or neuroretina of mice as described previously [27]. Briefly, eyes were collected and washed in cold PBS. The cornea and lens were removed, and the neuroretina was gently detached from the eyecup. Four cuts were made through the RPE/choroid/sclera, from the peripheral edges towards the optic nerve to flatten the tissue. The flattened RPE/choroid/sclera and neuroretina were transferred to a centrifuge tube containing 200 µl Trizol for ~ 30 min followed by gentle tapping to release RPE cells from the eyecup. The choroid/sclera tissue was removed, and the remaining pellets from RPE/choroid/sclera were labeled as RPE isolate and collected for RNA isolation. The RPE pellets and the neuroretina were homogenized separately using a pestle grinder, and RNA was purified using an RNeasy Micro kit (Qiagen). Isolated RNA (1 µg) was reverse transcribed using the High Capacity cDNA Reverse Transcription Kit (Applied Biosystems) and subjected to quantitative real-time PCR (QuantStudio 12K Flex Real-Time PCR System; Thermo Fisher Scientific [RRID SCR_021098]) using QuantiTect SYBR Green Master Mix (QIAGEN, Germantown, MD). The primer sequences are listed in Table S1. Mean cycle threshold (CT) values were calculated for both control and experimental samples. Changes in mRNA expression were normalized to GAPDH mRNA expression using the 2^−∆∆CT^ method [28].

### Western blot analysis

Retinas were isolated and homogenized in 200 µL extraction buffer, as previously described [27]. The protein concentration was measured by DC protein assay (BioRad) according to the manufacturer’s instructions. Retinal homogenates were combined with Laemmli buffer, boiled, and separated in Criterion Precast 4–20% gels (BioRad). Proteins were transferred to a polyvinylidene fluoride membrane, blocked with 5% milk in Tris-buffered saline with Tween 20, and probed with primary and secondary antibodies (Table S2). Blots were developed using Clarity ECL Substrate (BioRad) and imaged with a FluorChem E System (ProteinSimple). Western blot band intensities were quantified using ImageJ software and protein abundance was expressed relative to actin, which was used as an internal loading control.

### Immunofluorescent (IF) microscopy

For RPE whole mounts, eyes were fixed in 4% paraformaldehyde (PFA) for 30 min at room temperature, followed by removal of the cornea, lens, and neuroretina as described previously [27]. The RPE, choroid, and sclera were flattened as described above, followed by washing (PBS, 5 min, 3 ×) and blocking with 5% horse serum for 1 h at room temperature. Whole mounts were then labeled with anti-REDD1 and anti-RPE65 primary antibodies and fluorophore-tagged secondary antibodies (Table S2). Alternatively, retinal sections were prepared from whole eyes, as described previously [29]. To do so, following 30 min fixation in 4% PFA, eyes were washed with PBS and incubated at 4 °C in a 30% sucrose solution containing 0.05% sodium azide for 48 h. Eyes were then embedded in optimal cutting temperature compound, flash frozen, and 10 µm thin sections were obtained using a cryostat. Sections were fixed in 4% PFA for 10 min and permeabilized in PBS with 0.1% Triton-X-100. Sections were blocked with 10% normal horse serum for 1 h and labeled with primary and fluorophore-tagged secondary antibodies (Table S2). All sections and whole mounts were stained with 1 µM DAPI to visualize the nuclei, mounted with Fluoromount aqueous mounting media (Sigma-Aldrich), and imaged by confocal laser microscope (Leica SP8; Leica) with frame-stack sequential scanning. Images from each section (8 Z-stacks) were acquired using the corresponding laser with a capture time of 72 s (9 s/Z-stack). The image acquisition parameters remained consistent across all samples within each experiment. The fluorescence image intensities from the merged Z-stack image were quantified using ImageJ software with a constant threshold setting to eliminate background [30].

### Histology and morphometric analysis

Retinal cryosections (10 µm) were stained with H&E and used for morphometric analysis to evaluate RPE integrity and retinal layer thickness. Images of retinal sections were obtained, and the thickness of the total retina; inner retina (including ganglion cell layer, inner plexiform layer, and inner nuclear layer); outer retina (including outer plexiform layer, outer nuclear layer, photoreceptor inner segments/outer segments, and RPE); and outer nuclear layer (ONL) were measured using ImageJ software with a reference scale bar under the same conditions for all samples. Measurements were taken at equal distances from the optic nerve in all the sections. The average thickness of 5–10 measurements was manually obtained from each sample image by an observer masked to sample identity.

### TUNEL assays

Terminal deoxynucleotidyltransferase dUTP nick-end labeling (TUNEL) was performed on retinal cryosections using an in situ cell death fluorescein-detection kit (Promega), according to the manufacturer’s instructions. Immunofluorescence signals were imaged by confocal laser microscopy. TUNEL-positive cells were counted by an observer masked to sample identity and the values were averaged. TUNEL-positive cells were counted in the outer nuclear layer from at least 3 fields of view per eye from 3 to 5 mice per group.

### Reactive oxygen species measurement

Retinal reactive oxygen species (ROS) levels were measured as described previously using 2,7-dichlorodihydrofluorescein (DCF) staining [17]. Briefly, retinal cryosections were stained with 10 µM DCF for 60 min at 37 °C. Nuclei were stained with DAPI. DCF detection at 505 nm was visualized by confocal laser microscopy and quantified using ImageJ with a background threshold setting.

### Statistical analysis

All values are presented as mean ± SD. At least two independent experiments were carried out, and statistical analysis was performed by one-way ANOVA using GraphPad Prism software followed by Sidak’s test for multiple comparisons. Values of *p* < 0.05 were considered statistically significant in all analyses.

## Results

### NaIO_3_ promoted REDD1 protein abundance in the RPE and retina

**Fig. 1:**
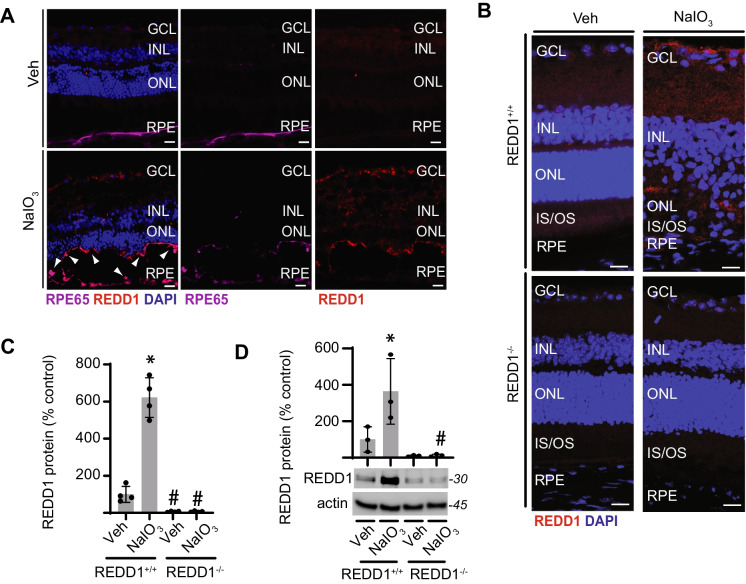
REDD1 protein abundance was increased in the retina of mice after NaIO_3_ administration. Mice were administered NaIO_3_ or saline vehicle (Veh) and retinas were analyzed 7 days later. **A** RPE65 (*purple*) and REDD1 (*red*) were visualized in retinal sections from wild-type mice by immunofluorescent microscopy (scale bar, 25 µm). Nuclei were counterstained with DAPI (*blue*). Arrowheads (*white*) indicate areas of REDD1 colocalization with RPE65. **B** REDD1 (*red*) protein was visualized in retinal sections from REDD1^+/+^ and REDD1^−/−^ mice. **C** REDD1 in **B** was quantified. **D** REDD1 protein abundance was evaluated in retinal homogenates by western blotting. Data presented include both male and female mice. Values are means ± SD (*n* = 3–4). Differences between groups were identified by one-way ANOVA. *, *p* ≤ 0.05 vs. Veh; #*p* ≤ 0.05 vs REDD1^+/+^; GCL, ganglion cell layer; INL, inner nuclear layer; ONL, outer nuclear layer; RPE, retinal pigment epithelium

REDD1 protein abundance in the retina was increased 7 days after administration of NaIO_3_ compared to vehicle control (Fig. 1A and Fig. S1). In mice receiving NaIO_3_, REDD1 was observed throughout the entire retina and partially co-localized with the RPE marker RPE65. The specificity of the REDD1 antibody was supported by the absence of signal in the retina of REDD1^−/−^ mice following NaIO_3_ administration (Fig. 1B, C). In support of the analysis by IF microscopy, western blotting also revealed increased REDD1 protein abundance in retinal homogenates from mice administered NaIO_3_ as compared to vehicle control (Fig. 1D). Together, the data provide evidence that REDD1 protein abundance is upregulated following NaIO_3_ administration.

### NaIO_3_-induced oxidative stress was attenuated in REDD1-deficient mice

**Fig. 2:**
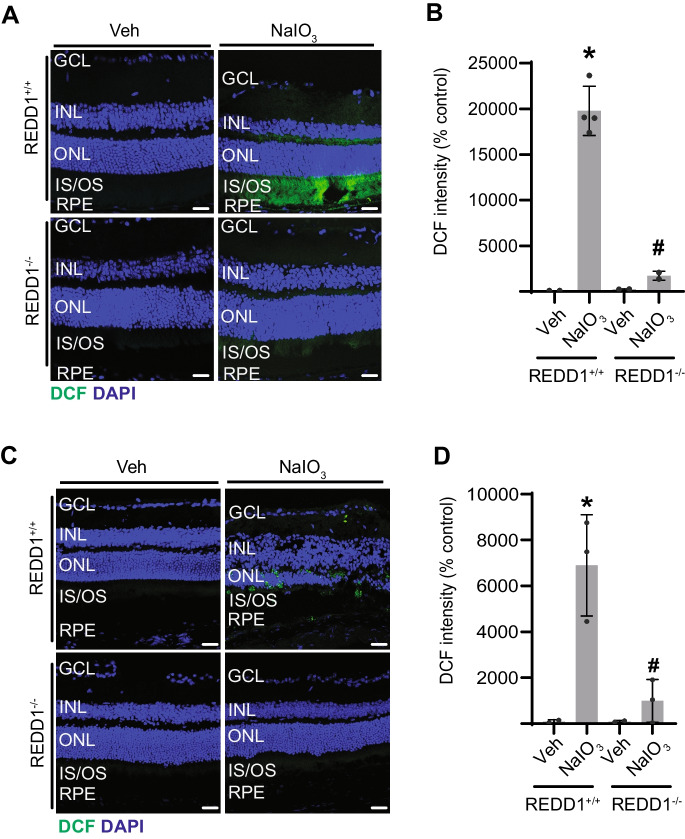
REDD1 deletion reduced oxidative stress in the retina of mice after NaIO_3_ administration. REDD1^+/+^ and REDD1^−/−^ mice were administered NaIO_3_ or saline vehicle (Veh). Reactive oxygen species were visualized in retinal sections using DCF either 1 (**A**, **B**) or 7 days (**C**, **D**) after NaIO_3_. DAPI was used to visualize nuclei. Representative images are shown in **A** and **C** (scale bar, 25 µm) with quantitative analysis in **B** and **D**, respectively. Data presented include both male and female mice. Values are means ± SD (*n* = 3–4). Differences between groups were identified by one-way ANOVA. *p ≤ 0.05 vs. Veh; #*p* ≤ 0.05 vs REDD1^+/+^; GCL, ganglion cell layer; INL, inner nuclear layer; ONL, outer nuclear layer; IS/OS, photoreceptor inner segments/outer segments; RPE, retinal pigment epithelium

The primary pathways underlying NaIO_3_-induced retinal damage are associated with the accumulation of ROS [31]. To determine the impact of REDD1 expression on NaIO_3_-induced oxidative stress, we quantified ROS levels in retinal sections by DCF staining. ROS levels were robustly increased in retinal sections from REDD1^+/+^ mice 1 day after NaIO_3_ administration. DCF fluorescence in retinal sections was predominantly localized throughout the RPE layer and photoreceptor inner and outer segments (IS/OS) (Fig. 2A, B). Seven days after NaIO_3_, ROS accumulation was observed in focal areas that localized throughout the outer retina (Fig. 2C, D). Importantly, ROS accumulation was substantially reduced in REDD1^−/−^ mice both at 1 and 7 days post-NaIO_3_ injection, as compared to REDD1^+/+^ mice. The data support a key role for REDD1 in the development of oxidative stress following NaIO_3_ administration.

### REDD1 deletion prevented NaIO_3_-induced immune cell activation in the retina

**Fig. 3:**
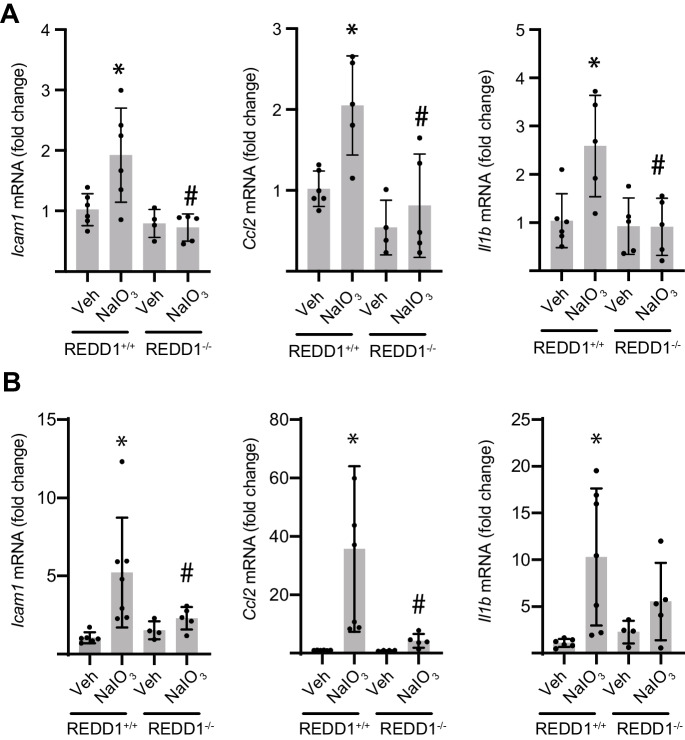
REDD1 deletion reduced NaIO_3_-induced immune signaling in RPE and retina. REDD1^+/+^ and REDD1^−/−^ mice were administered NaIO_3_ or saline vehicle (Veh). Gene expression was evaluated in RPE isolates (**A**) or neuroretina homogenate (**B**) 1 day after NaIO_3_. Data presented include both male and female mice. Values are means ± SD (*n* = 4–12). Differences between groups were identified by one-way ANOVA. *p ≤ 0.05 vs. Veh; #*p* ≤ 0.05 vs REDD1^+/+^

**Fig. 4:**
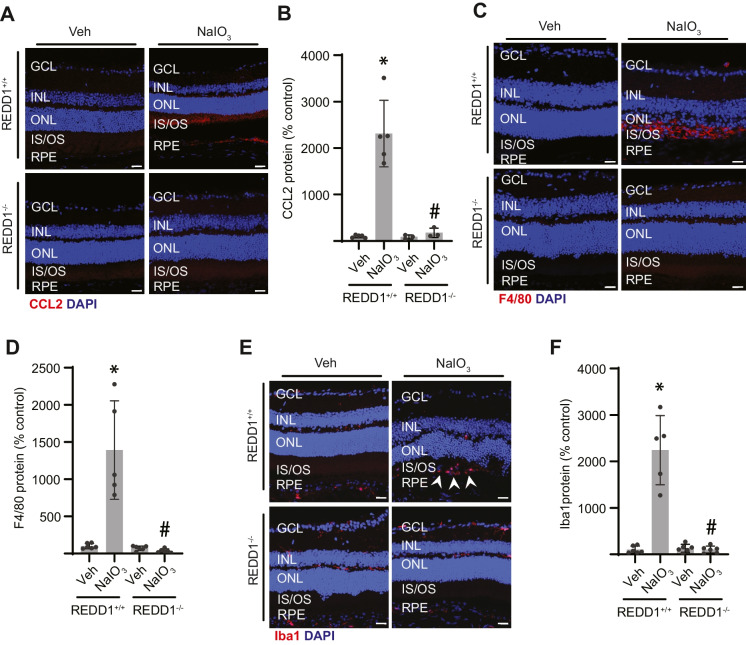
REDD1 deletion prevented NaIO_3_-induced immune cell activation in the retina. REDD1^+/+^ and REDD1^−/−^ mice were administered NaIO_3_ or saline vehicle (Veh). Seven days after NaIO_3_ administration, retinal sections were prepared and immunolabeled for CCL2 (**B**, *red*), F4/80 (**D**, *red*), or Iba1 (**F**, *red*) and counterstained with DAPI (*blue*). Representative images are shown (scale bar, 25 µm). Signal intensity for CCL2, F4/80, or Iba1 was quantified in **B**, **D,** and **F**, respectively. *White arrowheads* indicate the Iba1 + cells in the outer retina. Data presented include both male and female mice. Values are means ± SD (*n* = 3–5). Differences between groups were identified by one-way ANOVA. *p ≤ 0.05 vs. Veh; #*p* ≤ 0.05 vs REDD1^+/+^; GCL, ganglion cell layer; INL, inner nuclear layer; ONL, outer nuclear layer; IS/OS, photoreceptor inner segments/outer segments; RPE, retinal pigment epithelium

To investigate a role for REDD1 in NaIO_3_-induced retinal inflammation, we analyzed the expression of genes involved in immune signaling in the RPE and neuroretina. In RPE isolates from REDD1^+/+^ mice, *Icam1*, *Ccl2*, and *Il1b* mRNA expression were upregulated 1 day after NaIO_3_ administration (Fig. 3A). Increased expression of these pro-inflammatory markers following NaIO_3_ administration in the RPE isolate was largely resolved by 7 days (Fig. S3A). In the neuroretina of REDD1^+/+^ mice, *Icam1*, *Ccl2*, and *Il1b* mRNA expression were upregulated at both 1 (Fig. 3B) and 7 days (Fig. S3B) after NaIO_3_ injection. Remarkably, the deletion of REDD1 prevented the upregulation of *Icam1*, *Ccl2*, and *Il1b* mRNA expression in both RPE isolate and neuroretina after NaIO_3_ administration. Consistent with the NaIO_3_-induced increase in *Ccl2* mRNA, CCL2 (C–C motif chemokine ligand 2) protein was also increased in the RPE and photoreceptor IS/OS of REDD1^+/+^ mice at 7 days post-NaIO_3_, but not in REDD1^−/−^ mice (Fig. 4A, B). In the retina of REDD1^+/+^ mice, NaIO_3_ administration promoted immune cell activation in the outer retina, as evidenced by enhanced F4/80 (Fig. 4C, D) and Iba1 (Fig. 4E, F) immunoreactivity. However, immune cell activation was not observed in the retina of REDD1^−/−^ mice following NaIO_3_ administration. The data support a critical role for REDD1 in the development of retinal inflammation following NaIO_3_ administration.

### REDD1 was required for NaIO_3_-induced loss of RPE and photoreceptor markers

**Fig. 5:**
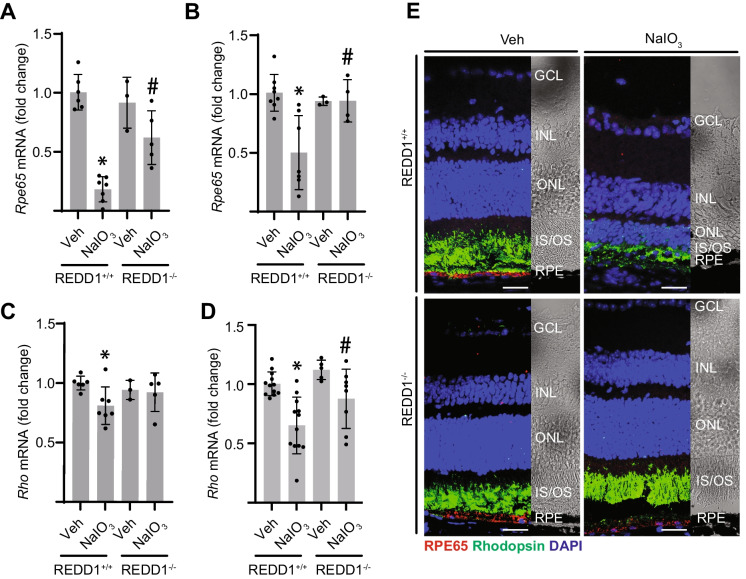
REDD1 was required for NaIO_3_-induced RPE and photoreceptor damage. REDD1^+/+^ and REDD1^−/−^ mice were administered NaIO_3_ or saline vehicle (Veh). **A**–**D** RT-PCR was used to quantify gene expression changes in RPE isolate (**A**, **B**) or neuroretinal homogenates (**C**, **D**). *Rpe65* expression was determined from the RPE isolate after 1 day (**A**) or 7 days (**B**) of post-NaIO_3_ injection. *Rho* gene expression was determined from neuroretina homogenate at 1 day (**C**) or 7 days (**D**) post-NaIO_3_ injection. **E** Representative immunostaining images of RPE65 and rhodopsin in retinal sections from REDD1^+/+^ and REDD1^−/−^ mice 7 days after NaIO_3_ administration (scale bar, 25 µm). Corresponding brightfield images are shown at the right. Nuclei were counterstained with DAPI. Data presented include both male and female mice. Values are means ± SD (*n* = 3–12). Differences between groups were identified by one-way ANOVA. *p ≤ 0.05 vs. Veh; #*p* ≤ 0.05 vs REDD1^+/+^; GCL, ganglion cell layer; INL, inner nuclear layer; ONL, outer nuclear layer; IS/OS, photoreceptor inner segments/outer segments; RPE, retinal pigment epithelium

In REDD1^+/+^ mice, expression of the RPE marker *Rpe65* in RPE isolate was reduced within 1 day of NaIO_3_ administration and this decrease remained up to 7 days (Fig. 5A, B). In the neuroretina, NaIO_3_ decreased expression of the photoreceptor marker *Rho* after 1 day, which became more severe after 7 days (Fig. 5C, D). Unlike REDD1^+/+^ mice, REDD1^−/−^ mice did not exhibit reduced expression of *Rpe65* or *Rho*. In support of the gene expression analysis, IF revealed decreased RPE65 in the RPE layer and reduced rhodopsin in the IS/OS 7 days after NaIO_3_ in REDD1^+/+^ mice, but not in REDD1^−/−^ mice (Fig. 5E).

### REDD1 deletion reduced NaIO_3_-induced retinal apoptosis and neurodegeneration

**Fig. 6:**
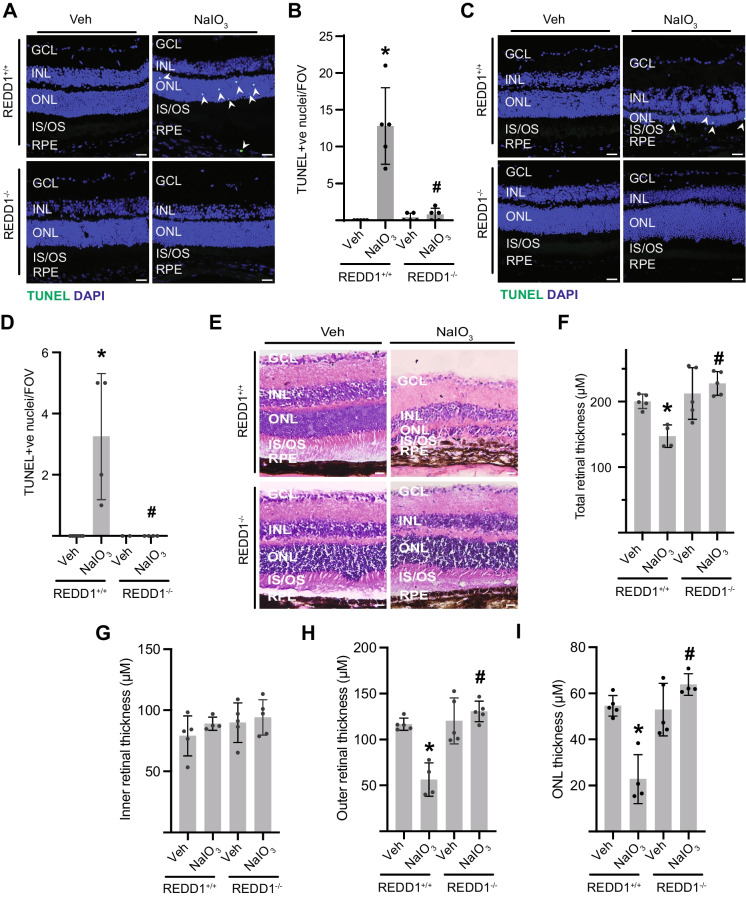
REDD1-deficient mice resisted NaIO_3_-induced retinal degeneration. REDD1^+/+^ and REDD1^−/−^ mice were administered NaIO_3_ or saline vehicle (Veh). **A**–**D** Cell death was evaluated in retinal sections by TUNEL staining either 1 (**A**, **B**) or 7 days (**C**, **D**) after NaIO_3_ administration. Representative images are shown (scale bar, 25 µm). TUNEL-positive nuclei per field of view (FOV, 900 µm^2^) in **A** and **C** were quantified in **B** and **D**, respectively. *White arrowheads* indicate TUNEL-positive cells. Retinal morphology, RPE-layer integrity, and loss of photoreceptors were evaluated by H&E-staining 7 days after NaIO_3_ administration. **F**–**I** Retinal layer thickness was quantified for total retina (**F**, GCL to RPE), inner retina (**G**, GCL to INL), outer retina (**H**, OPL to RPE), and ONL (**I**). Data presented include both male and female mice. Values are means ± SD (*n* = 4–5). Differences between groups were identified by one-way ANOVA. *p ≤ 0.05 vs. Veh; #*p* ≤ 0.05 vs REDD1^+/+^; GCL, ganglion cell layer; INL, inner nuclear layer; ONL, outer nuclear layer; IS/OS, photoreceptor inner segments/outer segments; RPE, retinal pigment epithelium

TUNEL staining was used to evaluate apoptosis in retinal cross-sections. NaIO_3_ increased TUNEL-positive nuclei in the RPE and ONL in REDD1^+/+^ mice within 1 day of administration (Fig. 6A, B). The increase in TUNEL-positive nuclei in the retina of REDD1^+/+^ mice was sustained 7 days after NaIO_3_ injection, at which time labeling was principally localized to the ONL (Fig. 6C, D). However, RPE and photoreceptor cell death was not observed in REDD1-deficient mice after NaIO_3_ administration. NaIO_3_ caused severe damage in the retinal of REDD1^+/+^ mice, characterized by the disrupted RPE layer integrity and photoreceptor degeneration (Fig. 6E). After NaIO_3_ treatment, total retinal thickness in the central area of the REDD1^+/+^ mice was reduced compared with the vehicle controls (Fig. 6F). NaIO_3_-induced retinal thinning was not observed in the inner retinal layer (Fig. 6G), as it was localized to the outer retinal layer (Fig. 6H). In particular, ONL thickness was reduced in REDD1^+/+^ mice after NaIO_3_ administration, compared to mice receiving vehicle (Fig. 6I). Notably, REDD1 deletion prevented the reduction in retinal thickness and preserved retinal morphology. Retinal morphology in vehicle-treated REDD1^+/+^ mice was not different from that in vehicle-treated REDD1^−/−^ mice.

## Discussion

In the present study, we examined a role for REDD1 in NaIO_3_-induced retinal injury in mice. Prior studies have administered NaIO_3_ to rats [32–34], mice [35, 36], rabbits [37, 38], cats [39], primates [40], and pigs [41] as an experimental model to study mechanisms that contribute to RPE and photoreceptor damage. Systemic administration of NaIO_3_ induces dose-dependent oxidative stress and selectively damages RPE [42, 43]. While NaIO_3_-induced is a helpful experimental model to study mechanisms of RPE damage, it is important to note its acute time course (days) versus the chronic disease progression that occurs in AMD (years). Consistent with AMD, NaIO_3_-induced RPE damage predominantly occurs in the central and middle regions of the retina and leads to subsequent photoreceptor degeneration [24]. Herein, NaIO_3_ administration was sufficient to increase REDD1 protein abundance throughout the entire retina. NaIO_3_ induced oxidative damage, retinal inflammation, and cell death in the RPE and photoreceptors. Importantly, REDD1 deletion protected RPE and photoreceptors from NaIO_3_-induced oxidative stress, decreased immune cell activation, and prevented retinal degeneration.

NaIO_3_ promoted REDD1 protein abundance throughout the entire retina with the increase partially localized to RPE. Notably, the increase in REDD1 protein abundance in response to NaIO_3_ was not accompanied by a corresponding increase in REDD1 mRNA expression in retinal homogenates (Fig. S2C). The observation aligns with our previous research indicating that increased ROS levels act to promote REDD1 protein abundance in the retina through a post-transcriptional mechanism [13]. Specifically, the formation of a reversible redox-sensitive disulfide bond prevents the normally rapid degradation of the REDD1 protein, leading to an increase in REDD1 protein abundance without an increase in REDD1 gene expression. The data here suggest that NaIO_3_ may act through a similar post-transcriptional mechanism to upregulate REDD1.

Our laboratory previously reported that retinal REDD1 is also upregulated by diabetes and is essential for the development of retinal defects and vision function deficits caused by diabetes [17, 44]. REDD1 is a stress-responsive protein[14], as it is upregulated by a range of cell stressors, including hypoxia, nutrient deprivation, endoplasmic reticular stress, and DNA damage [45]. Notably, oxidative stress and free radical formation are common to all of these cell stressors [14]. The primary pathways responsible for NaIO_3_-induced RPE/photoreceptor dysfunction are also associated with increased oxidative stress. REDD1 contributes to oxidative stress in the context of diabetes by both promoting ROS production and undermining the endogenous antioxidant response [15]. Extending on the prior report, we found that REDD1 was necessary for NaIO_3_-induced oxidative stress in RPE and photoreceptors. Together, the findings support a key role for REDD1 in the development of oxidative stress in the context of retinal disease.

While oxidative stress acts as an initiating mechanism, NaIO_3_-induced retinal degeneration is multifactorial, involving an upregulated inflammatory response and immune cell activation [23, 24, 46]. NaIO_3_ increased expression of both *Icam1*, which facilitates the adhesion of monocytes, and *Ccl2*, which serves as a chemoattractant for macrophages in the RPE and neuroretina [47, 48]. CCL2 levels and the subretinal infiltration of inflammatory monocytes are increased in the outer retina of patients with geographic atrophy and are believed to contribute to photoreceptor degeneration [49]. Prior studies indicate that macrophages migrate to and accumulate around melanin aggregations in the outer retina [23]. The role of CCL2 as a major factor in AMD pathogenesis is supported by reports showing that genetic deletion of CCL2 prevents inflammatory monocyte recruitment and photoreceptor degeneration in models of AMD [49]. Consistent with these reports, a single injection of NaIO_3_ enhanced Iba + and F4/80 + immune cells in the outer retina in a manner that was REDD1-dependent. Additionally, expression of the inflammatory cytokine IL1β, which is induced by activated immune cells, was increased in both RPE and neuroretina isolates 1 day after NaIO_3_ administration.

Infiltrating macrophages play a significant role in mediating photoreceptor cell death through the activation of inflammasomes [50]. Our previous findings have shown that REDD1 is involved in diabetes-induced macrophage infiltration, inflammasome activation, and inflammation in the retinas of diabetic mice [21, 29, 51]. Moreover, REDD1 is upregulated in response to lipopolysaccharide (LPS) and augments inflammatory responses [52–54]. The decreased activation and signaling of Iba1 + and F4/80 + cells observed in REDD1-deficient mice after administration of NaIO_3_ supports the presence of a REDD1-driven chemokine gradient that attracts these cells to the outer retina. Surprisingly, upregulated pro-inflammatory gene expression in RPE isolates in wild-type mice was largely resolved 7 days after NaIO_3_. An important caveat to this observation is that the collection of RPE isolates at this timepoint may not be equivalent to those in control retinas, as RPE layer integrity has become disrupted with cells potentially undergoing epithelial-to-mesenchymal transition or cell death. Thus, the remaining cells in the RPE isolate may be predominantly from the middle/peripheral retina, which is less affected by NaIO_3_ [55].

Healthy RPE cells express high levels of RPE65 [56], which plays a central role in the retinoid metabolic pathway, whereby RPE65 converts all-trans-retinyl ester to 11-cis-retinol in the visual cycle [57, 58]. NaIO_3_ selectively damages RPE, leading to a decrease in RPE65 that is associated with the loss of normal RPE characteristics and disrupted RPE layer integrity [24]. Herein, RPE65 levels were reduced in wild-type mice within 1 day of NaIO_3_ administration. RPE dysfunction was followed by detrimental effects on photoreceptors, as evidenced by decreased rhodopsin expression and collapse of the photoreceptor layer. Unlike wild-type mice, REDD1-deficient mice did not exhibit reduced RPE65 levels following NaIO_3_ administration and photoreceptor degeneration was absent. The observation supports a key role for REDD1 in the development of NaIO_3_-induced RPE dysfunction.

In the retinas of patients with AMD, apoptotic photoreceptors are observed near regions of RPE atrophy, suggesting that RPE damage may precede photoreceptor cell death during disease progression [59]. RPE and photoreceptor atrophy, accompanied by thinning of retinal layers, are associated with vision loss in both AMD patients [60] and mouse models of NaIO_3_ administration [22, 36]. NaIO_3_ is believed to selectively damage RPE cells, which subsequently results in photoreceptor loss [61]. One day after NaIO_3_ administration, many apoptotic nuclei were localized to the RPE and photoreceptor layers of wild-type mice, with relatively limited disruption of retinal morphology. Seven days after NaIO_3_ administration, we observed degeneration of the outer retina in wild-type mice, whereas the inner retinal layers were largely preserved. Loss of photoreceptors 7 days after NaIO_3_ administration was evidenced by reduced rhodopsin expression and thinning of the outer nuclear layer. Remarkably, REDD1 deletion prevented NaIO_3_-induced RPE atrophy and photoreceptor degeneration.

Dry AMD is characterized by progressive deterioration of RPE and photoreceptor cells in the macula, leading to central vision loss. The proof-of-concept studies herein suggest the possibility that therapeutics targeting REDD1 could potentially provide interventions in the dry stages of AMD by preventing RPE damage and photoreceptor loss. The principal evidence-based treatment for AMD is therapeutics that block vascular endothelial growth factor (VEGF). One of the limitations of this approach is that it largely addresses the microvascular dysfunction and neovascularization that characterize wet AMD. Indeed, REDD1 silencing was previously pursued as a therapeutic target for wet AMD, but the approach was ultimately abandoned over a decade ago due to inferiority to anti-VEGF [62]. While inferior to anti-VEGF, best corrected visual acuity (BCVA) was modestly improved in patients with wet AMD when treated intravitreally with a siRNA targeting the REDD1 mRNA (PF-04523655). An important caveat of these studies is that the efficacy of PF-04523655 for REDD1 suppression could not be determined in the eyes of patients. Notably, intravitreal PF-04523655 administration failed to suppress REDD1 mRNA expression in RPE/choroid isolates obtained from rats 14 days after laser injury to rupture Bruch’s membrane [63]. Thus, interventions that more effectively suppress REDD1 expression in RPE could potentially provide hope as a therapeutic in patients with dry AMD.

## Supplementary Information

Below is the link to the electronic supplementary material.

Supplementary file1 (PDF 850 KB)

## Data Availability

Primary data is available at 10.6084/m9.figshare.27145092. All other materials are available from the corresponding author upon request.

## References

[1] Fleckenstein M, Keenan TDL, Guymer RH, Chakravarthy U, Schmitz-Valckenberg S, Klaver CC, et al. Age-related macular degeneration. Nat Rev Dis Primers. 2021;7:31. 10.1038/s41572-021-00265-2. 33958600 10.1038/s41572-021-00265-2 PMC12878645

[2] Blasiak J, Sobczuk P, Pawlowska E, Kaarniranta K. Interplay between aging and other factors of the pathogenesis of age-related macular degeneration. Ageing Res Rev. 2022;81:101735. 10.1016/j.arr.2022.101735. 36113764 10.1016/j.arr.2022.101735

[3] Sharma R, Bose D, Maminishkis A, Bharti K. Retinal pigment epithelium replacement therapy for age-related macular degeneration: are we there yet? Annu Rev Pharmacol Toxicol. 2020;60:553–72. 10.1146/annurev-pharmtox-010919-023245. 31914900 10.1146/annurev-pharmtox-010919-023245 PMC8783375

[4] Finnemann SC. Focal adhesion kinase signaling promotes phagocytosis of integrin-bound photoreceptors. EMBO J. 2003;22:4143–54. 10.1093/emboj/cdg416. 12912913 10.1093/emboj/cdg416 PMC175805

[5] Lakkaraju A, Umapathy A, Tan LX, Daniele L, Philp NJ, Boesze-Battaglia K, et al. The cell biology of the retinal pigment epithelium. Prog Retin Eye Res. 2020;100846. 10.1016/j.preteyeres.2020.100846. 10.1016/j.preteyeres.2020.100846 PMC8941496 32105772

[6] Strauss O. The retinal pigment epithelium in visual function. Physiol Rev. 2005;85:845–81. 10.1152/physrev.00021.2004. 15987797 10.1152/physrev.00021.2004

[7] Simó R, Villarroel M, Corraliza L, Hernández C, Garcia-Ramírez M. The retinal pigment epithelium: something more than a constituent of the blood-retinal barrier–implications for the pathogenesis of diabetic retinopathy. J Biomed Biotechnol. 2010;2010:190724. 10.1155/2010/190724. 20182540 10.1155/2010/190724 PMC2825554

[8] Bazan NG. Survival signaling in retinal pigment epithelial cells in response to oxidative stress: significance in retinal degenerations. Adv Exp Med Biol. 2006;572:531–40. 10.1007/0-387-32442-9_74. 17249620 10.1007/0-387-32442-9_74

[9] Cruz-Guilloty F, Perez VL. Molecular medicine: defence against oxidative damage. Nature. 2011;478:42–3. 10.1038/478042a. 21979040 10.1038/478042a

[10] Ambati J, Fowler BJ. Mechanisms of age-related macular degeneration. Neuron. 2012;75:26–39. 10.1016/j.neuron.2012.06.018. 22794258 10.1016/j.neuron.2012.06.018 PMC3404137

[11] Shaw PX, Stiles T, Douglas C, Ho D, Fan W, Du H, et al. Oxidative stress, innate immunity, and age-related macular degeneration. AIMS Mol Sci. 2016;3:196–221. 10.3934/molsci.2016.2.196. 27239555 10.3934/molsci.2016.2.196 PMC4882104

[12] Shoshani T, Faerman A, Mett I, Zelin E, Tenne T, Gorodin S, et al. Identification of a novel hypoxia-inducible factor 1-responsive gene, RTP801, involved in apoptosis. Mol Cell Biol. 2002;22:2283–93. 10.1128/MCB.22.7.2283-2293.2002. 11884613 10.1128/MCB.22.7.2283-2293.2002 PMC133671

[13] Miller WP, Sha CM, Sunilkumar S, Toro AL, VanCleave AM, Kimball SR, et al. Activation of disulfide redox switch in REDD1 promotes oxidative stress under hyperglycemic conditions. Diabetes. 2022;71:2764–76. 10.2337/db22-0355. 36170669 10.2337/db22-0355 PMC9750946

[14] Miller WP, Sunilkumar S, Dennis MD. The stress response protein REDD1 as a causal factor for oxidative stress in diabetic retinopathy. Free Radic Biol Med. 2021;165:127–36. 10.1016/j.freeradbiomed.2021.01.041. 33524531 10.1016/j.freeradbiomed.2021.01.041 PMC7956244

[15] Miller WP, Sunilkumar S, Giordano JF, Toro AL, Barber AJ, Dennis MD. The stress response protein REDD1 promotes diabetes-induced oxidative stress in the retina by Keap1-independent Nrf2 degradation. J Biol Chem. 2020;295:7350–61. 10.1074/jbc.RA120.013093. 32295843 10.1074/jbc.RA120.013093 PMC7247303

[16] Dennis MD, Kimball SR, Fort PE, Jefferson LS. Regulated in development and DNA damage 1 is necessary for hyperglycemia-induced vascular endothelial growth factor expression in the retina of diabetic rodents. J Biol Chem. 2015;290:3865–74. 10.1074/jbc.M114.623058. 25548280 10.1074/jbc.M114.623058 PMC4319049

[17] Miller WP, Toro AL, Sunilkumar S, Stevens SA, VanCleave AM, Williamson DL, et al. Müller glial expression of REDD1 is required for retinal neurodegeneration and visual dysfunction in diabetic mice. Diabetes. 2022;71:1051–62. 10.2337/db21-0853. 35167652 10.2337/db21-0853 PMC9074768

[18] Miller WP, Toro AL, Barber AJ, Dennis MD. REDD1 activates a ROS-generating feedback loop in the retina of diabetic mice. Invest Ophthalmol Vis Sci. 2019;60:2369–79. 10.1167/iovs.19-26606. 31141608 10.1167/iovs.19-26606 PMC6541429

[19] Chen Q, Xin G, Li S, Dong Y, Yu X, Wan C, et al. Berberine-mediated REDD1 down-regulation ameliorates senescence of retinal pigment epithelium by interrupting the ROS-DDR positive feedback loop. Phytomedicine. 2022;104:154181. 10.1016/j.phymed.2022.154181. 35792445 10.1016/j.phymed.2022.154181

[20] Li R, Ye Z, Xu Y, Liu Y. Cyanidin-3-O-glucoside from blueberry anthocyanin extracts protects ARPE-19 cells against high glucose damage via REDD1/GSK3β pathway. Food Biosci. 2023;56:103322. 10.1016/j.fbio.2023.103322.

[21] Sunilkumar S, VanCleave AM, McCurry CM, Toro AL, Stevens SA, Kimball SR, et al. REDD1-dependent GSK3β dephosphorylation promotes NF-κB activation and macrophage infiltration in the retina of diabetic mice. J Biol Chem. 2023;299:104991. 10.1016/j.jbc.2023.104991. 37392853 10.1016/j.jbc.2023.104991 PMC10407432

[22] Espitia-Arias MD, de la Villa P, Paleo-García V, Germain F, Milla-Navarro S. Oxidative model of retinal neurodegeneration induced by sodium iodate: morphofunctional assessment of the visual pathway. Antioxidants (Basel). 2023;12:1594. 10.3390/antiox12081594. 37627589 10.3390/antiox12081594 PMC10451746

[23] Moriguchi M, Nakamura S, Inoue Y, Nishinaka A, Nakamura M, Shimazawa M, et al. Irreversible photoreceptors and RPE cells damage by intravenous sodium iodate in mice is related to macrophage accumulation. Invest Ophthalmol Vis Sci. 2018;59:3476–87. 10.1167/iovs.17-23532. 30025075 10.1167/iovs.17-23532

[24] Yang X, Rai U, Chung J-Y, Esumi N. Fine tuning of an oxidative stress model with sodium iodate revealed protective effect of NF-κB inhibition and sex-specific difference in susceptibility of the retinal pigment epithelium. Antioxidants (Basel). 2021;11:103. 10.3390/antiox11010103. 35052607 10.3390/antiox11010103 PMC8773095

[25] Brafman A, Mett I, Shafir M, Gottlieb H, Damari G, Gozlan-Kelner S, et al. Inhibition of oxygen-induced retinopathy in RTP801-deficient mice. Invest Ophthalmol Vis Sci. 2004;45:3796–805. 10.1167/iovs.04-0052. 15452091 10.1167/iovs.04-0052

[26] Anderson BD, Lee TT, Bell BA, Wang T, Dunaief JL. Optimizing the sodium iodate model: effects of dose, gender, and age. Exp Eye Res. 2024;239:109772. 10.1016/j.exer.2023.109772. 38158173 10.1016/j.exer.2023.109772 PMC10922497

[27] Wei H, Xun Z, Granado H, Wu A, Handa JT. An easy, rapid method to isolate RPE cell protein from the mouse eye. Exp Eye Res. 2016;145:450–5. 10.1016/j.exer.2015.09.015. 26424220 10.1016/j.exer.2015.09.015 PMC4809787

[28] Livak KJ, Schmittgen TD. Analysis of relative gene expression data using real-time quantitative PCR and the 2(-delta delta C(T)) method. Methods. 2001;25:402–8. 10.1006/meth.2001.1262. 11846609 10.1006/meth.2001.1262

[29] Sunilkumar S, Toro AL, McCurry CM, VanCleave AM, Stevens SA, Miller WP, et al. Stress response protein REDD1 promotes diabetes-induced retinal inflammation by sustaining canonical NF-κB signaling. J Biol Chem. 2022;298:102638. 10.1016/j.jbc.2022.102638. 36309088 10.1016/j.jbc.2022.102638 PMC9694114

[30] Shihan MH, Novo SG, Le Marchand SJ, Wang Y, Duncan MK. A simple method for quantitating confocal fluorescent images. Biochem Biophys Rep. 2021;25:100916. 10.1016/j.bbrep.2021.100916. 33553685 10.1016/j.bbrep.2021.100916 PMC7856428

[31] Liu Y, Li R, Xie J, Hu J, Huang X, Ren F, et al. Protective effect of hydrogen on sodium iodate-induced age-related macular degeneration in mice. Front Aging Neurosci. 2018;10:389. 10.3389/fnagi.2018.00389. 30564112 10.3389/fnagi.2018.00389 PMC6288204

[32] Koster C, van den Hurk KT, Ten Brink JB, Lewallen CF, Stanzel BV, Bharti K, et al. Sodium-iodate injection can replicate retinal degenerative disease stages in pigmented mice and rats: non-invasive follow-up using OCT and ERG. Int J Mol Sci. 2022;23:2918. 10.3390/ijms23062918. 35328338 10.3390/ijms23062918 PMC8953416

[33] Koh AE-H, Alsaeedi HA, Rashid MBA, Lam C, Harun MHN, Saleh MFBM, et al. Retinal degeneration rat model: a study on the structural and functional changes in the retina following injection of sodium iodate. J Photochem Photobiol B 2019;196:111514. 10.1016/j.jphotobiol.2019.111514. 10.1016/j.jphotobiol.2019.111514 31154277

[34] Chen S, Liu G, Liu X, Wang Y, He F, Nie D, et al. RNA-seq analysis reveals differentially expressed inflammatory chemokines in a rat retinal degeneration model induced by sodium iodate. J Int Med Res. 2022;50:3000605221119376. 10.1177/03000605221119376. 36036255 10.1177/03000605221119376 PMC9434683

[35] Hosoda L, Adachi-Usami E, Mizota A, Hanawa T, Kimura T. Early effects of sodium iodate injection on ERG in mice. Acta Ophthalmol (Copenh). 1993;71:616–22. 10.1111/j.1755-3768.1993.tb04650.x. 8109207 10.1111/j.1755-3768.1993.tb04650.x

[36] Chowers G, Cohen M, Marks-Ohana D, Stika S, Eijzenberg A, Banin E, et al. Course of sodium iodate-induced retinal degeneration in albino and pigmented mice. Invest Ophthalmol Vis Sci. 2017;58:2239–49. 10.1167/iovs.16-21255. 28418497 10.1167/iovs.16-21255

[37] Jang K-H, Do Y-J, Koo T-S, Choi J-S, Song EJ, Hwang Y, et al. Protective effect of RIPK1-inhibitory compound in in vivo models for retinal degenerative disease. Exp Eye Res. 2019;180:8–17. 10.1016/j.exer.2018.11.026. 30500363 10.1016/j.exer.2018.11.026

[38] Cho B-J, Seo J-M, Yu HG, Chung H. Monocular retinal degeneration induced by intravitreal injection of sodium iodate in rabbit eyes. Jpn J Ophthalmol. 2016;60:226–37. 10.1007/s10384-016-0429-1. 26902976 10.1007/s10384-016-0429-1

[39] Kiryu J, Yamamoto F, Honda Y. Effects of sodium iodate on the electroretinogram c-wave in the cat. Vision Res. 1992;32:2221–7. 10.1016/0042-6989(92)90086-x. 1287999 10.1016/0042-6989(92)90086-x

[40] Ou Q, Zhu T, Li P, Li Z, Wang L, Lian C, et al. Establishment of retinal degeneration model in rat and monkey by intravitreal injection of sodium iodate. Curr Mol Med. 2018;18:352–64. 10.2174/1566524018666181113104023. 30421676 10.2174/1566524018666181113104023

[41] Monés J, Leiva M, Peña T, Martínez G, Biarnés M, Garcia M, et al. A swine model of selective geographic atrophy of outer retinal layers mimicking atrophic AMD: a phase I escalating dose of subretinal sodium iodate. Invest Ophthalmol Vis Sci. 2016;57:3974–83. 10.1167/iovs.16-19355. 27479813 10.1167/iovs.16-19355

[42] Machalińska A, Lubiński W, Kłos P, Kawa M, Baumert B, Penkala K, et al. Sodium iodate selectively injuries the posterior pole of the retina in a dose-dependent manner: morphological and electrophysiological study. Neurochem Res. 2010;35:1819–27. 10.1007/s11064-010-0248-6. 20725778 10.1007/s11064-010-0248-6 PMC2957578

[43] Kiuchi K, Yoshizawa K, Shikata N, Moriguchi K, Tsubura A. Morphologic characteristics of retinal degeneration induced by sodium iodate in mice. Curr Eye Res. 2002;25:373–9. 10.1076/ceyr.25.6.373.14227. 12789545 10.1076/ceyr.25.6.373.14227

[44] Miller WP, Yang C, Mihailescu ML, Moore JA, Dai W, Barber AJ, et al. Deletion of the Akt/mTORC1 repressor REDD1 prevents visual dysfunction in a rodent model of type 1 diabetes. Diabetes. 2018;67:110–9. 10.2337/db17-0728. 29074598 10.2337/db17-0728 PMC5741149

[45] Qiao S, Dennis M, Song X, Vadysirisack DD, Salunke D, Nash Z, et al. A REDD1/TXNIP pro-oxidant complex regulates ATG4B activity to control stress-induced autophagy and sustain exercise capacity. Nat Commun. 2015;6:7014. 10.1038/ncomms8014. 25916556 10.1038/ncomms8014 PMC4421852

[46] Mao X, Pan T, Shen H, Xi H, Yuan S, Liu Q. The rescue effect of mesenchymal stem cell on sodium iodate-induced retinal pigment epithelial cell death through deactivation of NF-κB-mediated NLRP3 inflammasome. Biomed Pharmacother. 2018;103:517–23. 10.1016/j.biopha.2018.04.038. 29677537 10.1016/j.biopha.2018.04.038

[47] Deshmane SL, Kremlev S, Amini S, Sawaya BE. Monocyte chemoattractant protein-1 (MCP-1): an overview. J Interferon Cytokine Res. 2009;29:313–26. 10.1089/jir.2008.0027. 19441883 10.1089/jir.2008.0027 PMC2755091

[48] Bui TM, Wiesolek HL, Sumagin R. ICAM-1: a master regulator of cellular responses in inflammation, injury resolution, and tumorigenesis. J Leukoc Biol. 2020;108:787–99. 10.1002/JLB.2MR0220-549R. 32182390 10.1002/JLB.2MR0220-549R PMC7977775

[49] Sennlaub F, Auvynet C, Calippe B, Lavalette S, Poupel L, Hu SJ, et al. CCR2(+) monocytes infiltrate atrophic lesions in age-related macular disease and mediate photoreceptor degeneration in experimental subretinal inflammation in Cx3cr1 deficient mice. EMBO Mol Med. 2013;5:1775–93. 10.1002/emmm.201302692. 24142887 10.1002/emmm.201302692 PMC3840491

[50] Kataoka K, Matsumoto H, Kaneko H, Notomi S, Takeuchi K, Sweigard JH, et al. Macrophage- and RIP3-dependent inflammasome activation exacerbates retinal detachment-induced photoreceptor cell death. Cell Death Dis. 2015;6:e1731. 10.1038/cddis.2015.73. 25906154 10.1038/cddis.2015.73 PMC4650542

[51] McCurry CM, Sunilkumar S, Subrahmanian SM, Yerlikaya EI, Toro AL, VanCleave AM, et al. NLRP3 inflammasome priming in the retina of diabetic mice requires REDD1-dependent activation of GSK3β. Invest Ophthalmol Vis Sci. 2024;65:34. 10.1167/iovs.65.3.34. 10.1167/iovs.65.3.34 PMC10981446 38546584

[52] Pastor F, Dumas K, Barthélémy M-A, Regazzetti C, Druelle N, Peraldi P, et al. Implication of REDD1 in the activation of inflammatory pathways. Sci Rep. 2017;7:7023. 10.1038/s41598-017-07182-z. 28765650 10.1038/s41598-017-07182-z PMC5539207

[53] Lee D-K, Kim J-H, Kim J, Choi S, Park M, Park W, et al. REDD-1 aggravates endotoxin-induced inflammation via atypical NF-κB activation. FASEB J. 2018;32:4585–99. 10.1096/fj.201701436R. 29547704 10.1096/fj.201701436R

[54] Lee D-K, Kim J-H, Kim W-S, Jeoung D, Lee H, Ha K-S, et al. Lipopolysaccharide induction of REDD1 is mediated by two distinct CREB-dependent mechanisms in macrophages. FEBS Lett. 2015;589:2859–65. 10.1016/j.febslet.2015.08.004. 26296313 10.1016/j.febslet.2015.08.004

[55] Zhang N, Zhang X, Girardot PE, Chrenek MA, Sellers JT, Li Y, et al. Electrophysiologic and morphologic strain differences in a low-dose NaIO_3_-induced retinal pigment epithelium damage model. Transl Vis Sci Technol. 2021;10:10. 10.1167/tvst.10.8.10. 10.1167/tvst.10.8.10 PMC8287050 34251426

[56] Hamel CP, Tsilou E, Pfeffer BA, Hooks JJ, Detrick B, Redmond TM. Molecular cloning and expression of RPE65, a novel retinal pigment epithelium-specific microsomal protein that is post-transcriptionally regulated in vitro. J Biol Chem. 1993;268:15751–7. 8340400

[57] Qtaishat NM, Redmond TM, Pepperberg DR. Acute radiolabeling of retinoids in eye tissues of normal and rpe65-deficient mice. Invest Ophthalmol Vis Sci. 2003;44:1435–46. 10.1167/iovs.02-0679. 12657577 10.1167/iovs.02-0679

[58] Katz ML, Wendt KD, Sanders DN. RPE65 gene mutation prevents development of autofluorescence in retinal pigment epithelial phagosomes. Mech Ageing Dev. 2005;126:513–21. 10.1016/j.mad.2004.11.004. 15722110 10.1016/j.mad.2004.11.004

[59] Dunaief JL, Dentchev T, Ying G-S, Milam AH. The role of apoptosis in age-related macular degeneration. Arch Ophthalmol. 2002;120:1435–42. 10.1001/archopht.120.11.1435. 12427055 10.1001/archopht.120.11.1435

[60] Horani M, Mahmood S, Aslam TM. A review of macular atrophy of the retinal pigment epithelium in patients with neovascular age-related macular degeneration: what is the link? Part II Ophthalmol Ther. 2020;9:35–75. 10.1007/s40123-019-00227-8. 31907843 10.1007/s40123-019-00227-8 PMC7054566

[61] Upadhyay M, Bonilha VL. Regulated cell death pathways in the sodium iodate model: insights and implications for AMD. Exp Eye Res. 2024;238:109728. 10.1016/j.exer.2023.109728. 37972750 10.1016/j.exer.2023.109728 PMC10841589

[62] Nguyen QD, Schachar RA, Nduaka CI, Sperling M, Klamerus KJ, Chi-Burris K, et al. Evaluation of the siRNA PF-04523655 versus ranibizumab for the treatment of neovascular age-related macular degeneration (MONET Study). Ophthalmology. 2012;119:1867–73. 10.1016/j.ophtha.2012.03.043. 22683252 10.1016/j.ophtha.2012.03.043

[63] Rittenhouse KD, Johnson TR, Vicini P, Hirakawa B, Kalabat D, Yang AH, et al. RTP801 gene expression is differentially upregulated in retinopathy and is silenced by PF-04523655, a 19-Mer siRNA directed against RTP801. Invest Ophthalmol Vis Sci. 2014;55:1232–40. 10.1167/iovs.13-13449. 24458146 10.1167/iovs.13-13449

