# Supplementary material for: Deletion of the stress response protein REDD1 prevents sodium iodate-induced RPE damage and photoreceptor loss
Source: GeroScience. 2024 Oct 5;47(2):1789–803. doi: 10.1007/s11357-024-01362-2 (PMC11979080; doi:10.1007/s11357-024-01362-2)
Supplement: Supplementary file 1 — Supplementary file1 (PDF 850 KB) [file 11357_2024_1362_MOESM1_ESM.pdf]

Supplemental Materials-

Deletion of the stress response protein REDD1 prevents sodium iodate-induced RPE damage and photoreceptor loss

Sandeep M. Subrahmanian[1], Esma I. Yerlikaya[1], Siddharth Sunilkumar[1], Allyson L. Toro[1], Christopher M. McCurry[1], Stephanie L. Grillo[2], Alistair J. Barber[1,2], Jeffrey M. Sundstrom[1,2], and Michael D. Dennis[1,2]

[1]Department of Cellular and Molecular Physiology, Penn State College of Medicine, Hershey, Pennsylvania 17033

[2]Department of Ophthalmology, Penn State College of Medicine, Hershey, Pennsylvania 17033

Table of Contents:

1. Table S1: Primers used for gene expression analysis

2. Table S2: Antibodies used for western blotting and immunofluorescence

3. Figure S1: Time-dependent changes in *Rpe65* and *Rho* expression after $NaIO_3$ administration

4. Figure S2: REDD1 protein abundance was increased in the retina of mice after $NaIO_3$ administration

5. Figure S3: REDD1 deletion reduced NaIO3-induced immune signaling in the RPE and retina

Table S1. Primers used for gene expression analysis

Gene	Primer sequence (5ˊ-3ˊ)	
	Forward	Reverse
REDD1	GGGATCGTTTCTCGTCCTCC	ATGAGGAGTCTTCCTCCGGC
Rpe65	TCTTACAGAGCTTGTCAGGAACA	CTCTTTTCAGGGCCTCGTCA
Rho	AGCAGCAGGCAGCCACC	CCGAAGTTGGAGCCCTGGTG
Icam1	AGCCTCCGGACTTCGATCT	TGTTTGTGCTCTCCTGGGTC
Ccl2	CACTCACCTGCTGCTACTCA	GCTTGGTGACAAAAACTACAGC
Il1β	CACTACAGGCTCCGAGATGAAC	CCCCTGGAGATTGAGCTGTCTGC
GAPDH	GGTGGTCTCCTCTGACTTCAACA	GTTGCTGTAGCCAAATTCGTTGT

Table S2. Antibodies used for western blotting and immunofluorescence

Assay	Antibody	Company	Catalogue	Lot	Dilution
Western Blot	REDD1	ProteinTech	10638-1-AP	00110532	1:500
	Actin	Cell signaling	4970	19	1:1000
Immunofluorescence	REDD1	ProteinTech	10638-1-AP	00110532	1:300
	RPE65	PETLET Rabbit monoclonal antibody from Dr. Michael Redmond, NIH-NEI			1:500
	RPE65 (Used for co-localization with REDD1)	Santacruz	sc-390787	K0522	1:500
	Rhodopsin		sc-57432	A1023	1:500
	CCL2	Novus	NBP1-07035AF532	D135527	1:200
	F4/80	Cell signaling	70076	8	1:250
	Iba1	Cell signaling	78060	2	1:100
	Donkey anti-Rabbit Alexafluor 488	Jackson ImmunoResearch	711-545-152	164289	1:800
	Donkey anti-Mouse Alexafluor 488		715-546-151	125414	1:800
	Donkey anti-Rabbit Alexafluor 594		711-585-152	164523	1:800
	Donkey anti-Mouse Alexafluor 647		715-605-150	163834	1:800

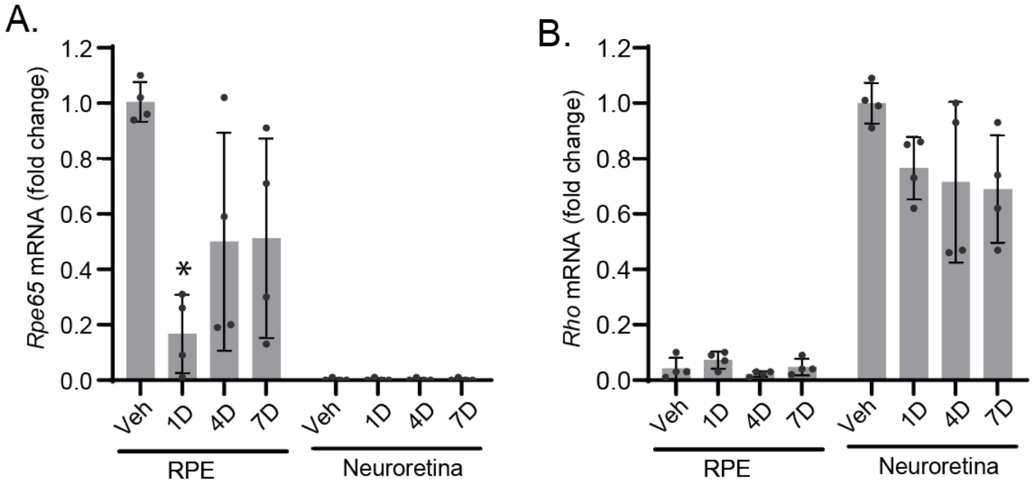

Figure S1. Time-dependent changes in *Rpe65* and *Rho* expression after $NaIO_3$ administration. Wild-type mice were administered either 20 mg/kg $NaIO_3$ or saline vehicle (Veh), and RPE and neuroretina isolates were collected for gene expression analysis after 1, 4, or 7 days. *Rpe65* gene expression (A) and *Rho* gene expression (B) were determined from both RPE isolate and neuroretina homogenate. Data presented include both male and female mice. Values are means + SD (n=4). *, $p \leq 0.05$ vs. Veh.

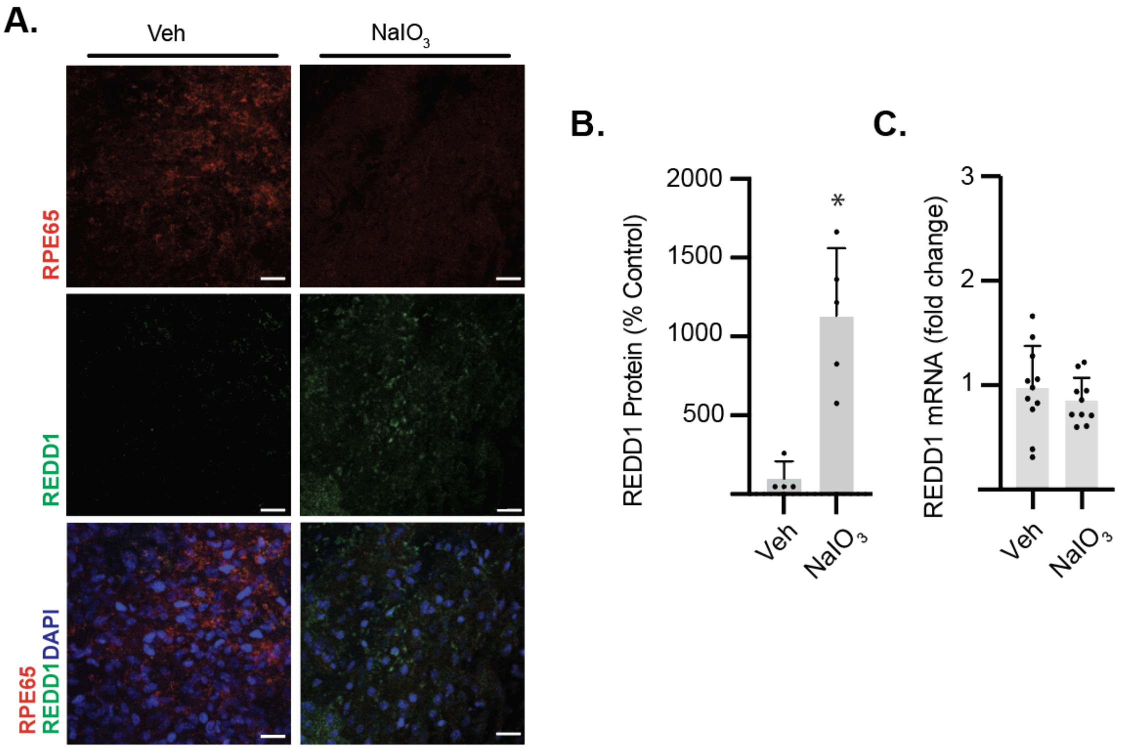

Figure S2. REDD1 protein abundance was increased in the RPE of mice after $NaIO_3$ administration. Mice were administered $NaIO_3$ or saline vehicle (Veh) and RPE were analyzed 7 days later. A, RPE65 (*red*) and REDD1 (*green*) were visualized in RPE whole mounts from wild-type mice by immunofluorescent microscopy (scale bar: 25 µm). Nuclei were counterstained with DAPI (*blue*). *B*, REDD1 protein was quantified in A. *C*, REDD1 mRNA abundance was quantified in retinal homogenates by RT-PCR. Data presented include both male and female mice. Values are means + SD (n=5-11). *, $p \leq 0.05$ vs. Veh.

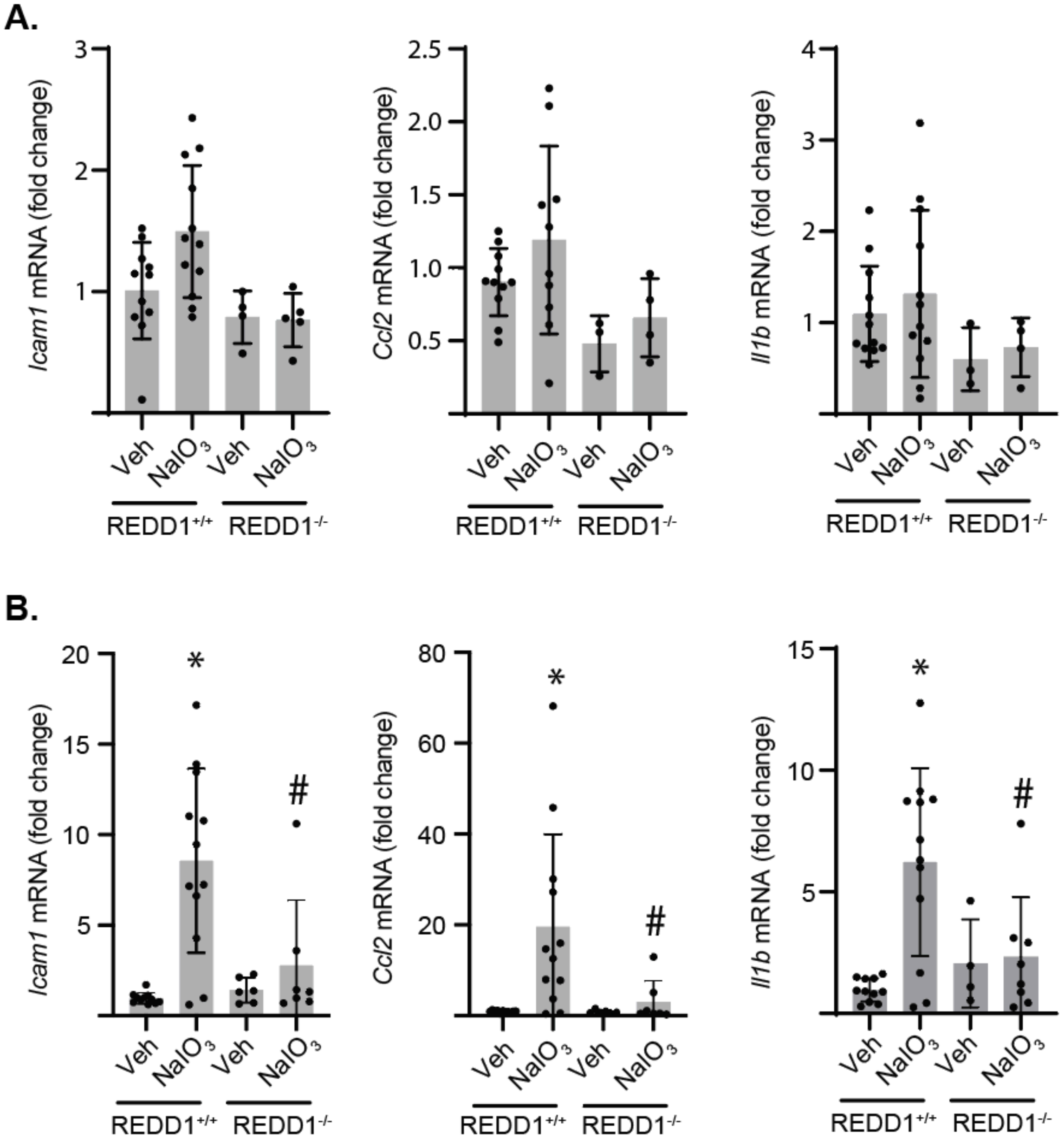

Figure S3. REDD1 deletion reduced $NaIO_3$-induced immune signaling in the RPE and retina. Expression levels of genes involved in immune signaling were measured in the RPE isolate (A) and neuroretina isolate by qRT-PCR from wild-type ($REDD1^{+/+}$) and REDD1 knockout ($REDD1^{-/-}$) mice at 7-day post $NaIO_3$ injection. Data presented include both male and female mice. Values are means ± SD (n=4-7). *P ≤ 0.05 vs. Veh; #P ≤ 0.05 vs $REDD1^{+/+}$.
